# Epigenetic Alterations in Hepatocellular Carcinoma: Mechanisms and Biomarkers for Precision Therapy

**DOI:** 10.3390/cancers18142224

**Published:** 2026-07-10

**Authors:** Binru Cai, Duoduo Lv, Qiang Qiu, Wenju Xiong, Heyu Tang, Yixiao Bai, Sicheng Zhou, Yiguo Hu, Rifaat Safadi, Chengdi Wang, Lingyun Zhou

**Affiliations:** 1 Center of Infectious Diseases, West China Hospital of Sichuan University, Chengdu 610041, China; caibinru@stu.scu.edu.cn (B.C.); lvduoduo@scu.edu.cn (D.L.); bai99417@163.com (Y.B.); 2025224025254@stu.scu.edu.cn (S.Z.); 2 Department of Hematology, State Key Laboratory of Biotherapy and Cancer Center, West China Hospital, Sichuan University, Chengdu 610041, China; qiuqiang@wchscu.cn; 3 Department of Head and Neck Oncology, State Key Laboratory of Biotherapy and Cancer Center, West China Hospital, Sichuan University, Chengdu 610041, China; xiongwenju@stu.scu.edu.cn; 4 Division of Cellular and Molecular Therapy, Department of Pediatrics, University of Florida, Gainesville, FL 32611, USA; heyu.tang@ufl.edu; 5 State Key Laboratory of Biotherapy and Cancer Center, West China Hospital, Sichuan University, Chengdu 610041, China; huyiguo@scu.edu.cn; 6 The Liver Institute, Hadassah Medical Organization, Hadassah Hebrew University Medical Center, P.O. Box 12000, Jerusalem 91120, Israel; safadi@hadassah.org.il; 7 Department of Respiratory and Critical Care Medicine, West China Hospital, Sichuan University, Chengdu 610041, China

**Keywords:** hepatocellular carcinoma, epigenetic alterations, noncoding RNAs, biomarkers, epigenetic therapy

## Abstract

Hepatocellular carcinoma (HCC), a leading cause of cancer-related mortality worldwide, is driven by complex interactions between genetic mutations and reversible epigenetic alterations. Among these, aberrant DNA methylation, histone modifications, and dysregulated noncoding RNAs (ncRNAs) play central roles in hepatocarcinogenesis, tumor progression, and therapy resistance. Epigenetic changes not only regulate key oncogenic pathways, including JAK/STAT and RAS, but also contribute to tumor immune evasion and heterogeneity. Unlike genetic mutations, epigenetic alterations are reversible, offering unique opportunities for therapeutic targeting. This review highlights recent advances in understanding the epigenetic landscape of HCC, identifies promising biomarkers for early detection and prognosis, and evaluates emerging epigenetic therapies (including DNMT, HDAC, and BET inhibitors as well as ncRNA-based strategies). Although these therapies have shown tumor-suppressive or treatment-sensitizing effects in preclinical models, their clinical translation remains limited by modest efficacy, small early-phase trials, treatment-related adverse events, and insufficient biomarker-guided patient selection. These insights may support more precise diagnostic and therapeutic strategies for HCC.

## Simple Summary

Hepatocellular carcinoma (HCC) remains one of the major contributors to cancer-related mortality worldwide, with most patients diagnosed at advanced stages with limited treatment options. Beyond genetic mutations, HCC development is strongly driven by reversible epigenetic alterations—heritable changes in gene expression that do not modify DNA sequences. This review synthesizes current evidence on how core epigenetic mechanisms, including DNA methylation, histone modifications, and noncoding RNA dysregulation, promote HCC tumor growth, metastasis, immune evasion, and drug resistance. It also highlights emerging epigenetic biomarkers for non-invasive early detection and prognosis, with relatively stronger evidence for DNA methylation- and hydroxymethylation-based markers. In addition, this review discusses epigenetic-targeted therapies and combination strategies, while acknowledging that most therapeutic evidence remains preclinical or early clinical. A better understanding of epigenetic mechanisms may support improved risk stratification, earlier diagnosis, and more precise treatment development for HCC.

## 1. Introduction

In 2022, liver cancer was the sixth most diagnosed cancer worldwide and the third leading cause of cancer-related death [1]. Hepatocellular carcinoma (HCC) constitutes the largest proportion of primary liver cancers, accounting for nearly 75–85% of cases and the majority of liver cancer–related deaths [2]. More than 90% of HCC cases result from chronic liver disease (CLD), particularly hepatitis B virus (HBV) or hepatitis C virus (HCV) infection [3], metabolic dysfunction-associated steatotic liver disease (MASLD) [4,5,6], and alcohol-associated liver disease (ALD) [7,8]. However, not all patients with CLD eventually develop cirrhosis or HCC, and the risk varies substantially across etiologies. For example, among patients with chronic hepatitis B (CHB) who do not receive antiviral therapy, the annual incidence of cirrhosis has been reported to be approximately 2–10%, whereas 3–6% of patients with HBV-related cirrhosis develop HCC each year [9]. Globally, an estimated 28% of cirrhosis cases and 26% of HCC cases are attributable to HCV infection [10]. In MASLD, progression to cirrhosis occurs in only a minority of patients, approximately 3–5%, and often takes more than 20 years [11]. Overall, patients with cirrhosis have an annual HCC incidence of approximately 1–6% [12]. At the molecular level, HCC initiation and progression are driven by the combined effects of genetic alterations; genomic instability; telomere dysfunction; dysregulated oncogenic signaling pathways, such as JAK/STAT and RAS signaling; and epigenetic remodeling. Representative events include the activation of oncogenes (such as *CTNNB1*, *MYC*, *PIK3CA*, *CCND1*, *TERT*, etc.) and the inactivation of tumor suppressor genes (TSGs) (such as *TP53*, *AXIN1*, *RB1*, *ARID1A*, *ARID2*, *PTEN*, etc.) [13,14,15,16,17]. Epigenetic dysregulation involves mechanisms such as DNA methylation, histone modifications, and ncRNAs regulation [18,19,20]. Overall, HCC initiation and progression are complex multistep processes involving genetic alterations, metabolic disorders, chronic inflammation, immune escape, and epigenetic remodeling. This review summarizes recent advances in key epigenetic mechanisms related to HCC tumorigenesis and progression. Although therapeutic strategies for HCC have improved substantially in recent decades, including interventional therapy, surgical resection, liver transplantation, targeted therapy, and immunotherapy [21], patient outcomes remain poor [22], underscoring the need for novel diagnostic and therapeutic strategies. Consequently, this review also summarizes epigenetic biomarkers and epigenetic-targeted therapies for HCC.

## 2. Epigenetic Alterations in HCC

**Figure 1:**
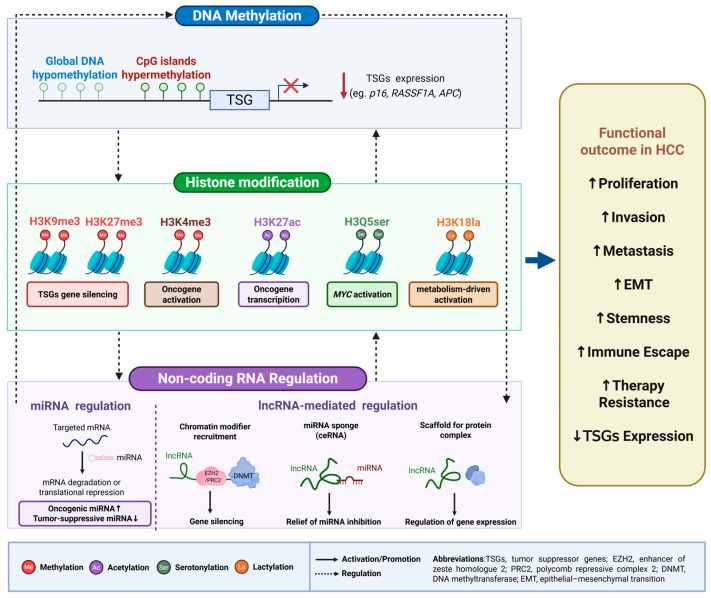
Integrated epigenetic regulation in HCC. DNA methylation, histone modifications, and noncoding RNA regulation jointly reshape gene-expression programs during HCC development. Aberrant DNA methylation contributes to TSG repression, while altered histone marks regulate chromatin accessibility and transcriptional activity. Noncoding RNAs modulate gene expression through miRNA-mediated post-transcriptional repression and lncRNA-mediated chromatin or RNA regulatory mechanisms. These epigenetic layers can also regulate each other. Together, these interconnected alterations promote malignant phenotypes, including proliferation, invasion, metastasis, EMT, stemness, immune escape, therapy resistance, and reduced TSG expression. Created in BioRender. Zhou, L. (2026) https://BioRender.com/2hqcdyx (accessed on 2 July 2026).

Epigenetic modifications are largely mediated by covalent modifications of histone proteins and nucleic acids, which cooperatively regulate chromatin structure and gene expression without DNA sequence alterations [23]. Epigenetic modifications participate in essential biological processes, including cell differentiation and embryogenesis. In cancer, including HCC, aberrant epigenetic alterations can reshape transcriptional programs, promote transcriptomic heterogeneity, and provide malignant cells with survival advantages during tumor progression [24,25]. Major epigenetic mechanisms include chromatin remodeling, DNA methylation, histone modification, and the regulation of ncRNA expression (Figure 1). These processes are closely implicated in HCC progression and metastatic dissemination.

### 2.1. DNA Methylation in HCC

Abnormal DNA methylation changes, including genome-wide loss of methylation and focal hypermethylation, are commonly detected in tumors and constitute hallmark epigenetic changes throughout the multistep process of carcinogenesis, including the development of HCC [26,27,28,29]. DNA methylation mainly occurs at cytosines within cytosine–guanine dinucleotides, also called cytosine–phosphate–guanine (CpG) sites. CpG sites are enriched in CpG islands, which are commonly found in the regulatory regions of more than half of human genes [30]. Promoter-associated CpG island hypermethylation is frequently observed in cancer cells and was first identified as a mechanism underlying the silencing of TSGs [31]. DNA methylation is dynamically regulated by enzymes responsible for methylation establishment, removal, and maintenance. DNMT3A and DNMT3B catalyze de novo methylation. TET1, TET2, and TET3 promote demethylation, and DNMT1 preserves existing methylation patterns during DNA replication [32].

In HCC, aberrant DNA methylation is considered an early pathogenic event that contributes to chromosomal instability [33]. In HBV-related HCC, hepatitis B virus x protein (HBx) promotes methylation dysregulation by enhancing overall DNA methyltransferase (DNMT) activity and inducing regional hypermethylation of selected TSGs. A representative example is E-cadherin, whose promoter CpG hypermethylation is a major mechanism responsible for its transcriptional silencing and has been reported in various malignancies, including HCC [34]. HBx can also directly interact with DNMT3A and recruit it to the promoter regions of *IL4R* and *MT1F*, leading to their transcriptional repression through de novo DNA methylation [35]. By repressing DNMT3B, HBx contributes to hypomethylation of satellite 2 repeat sequences at the global level. In HBV-associated HCC, these methylation abnormalities are closely linked to HBx expression [36]. HCV infection also affects DNA methylation in HCC. In the presence of HCV core protein, DNMT1 and DNMT3B cooperate to promote E-cadherin promoter methylation. As a key cell adhesion molecule and potential suppressor of invasion and metastasis, E-cadherin is transcriptionally downregulated by HCV core protein, which may contribute to hepatocarcinogenesis [37].

**Figure 2:**
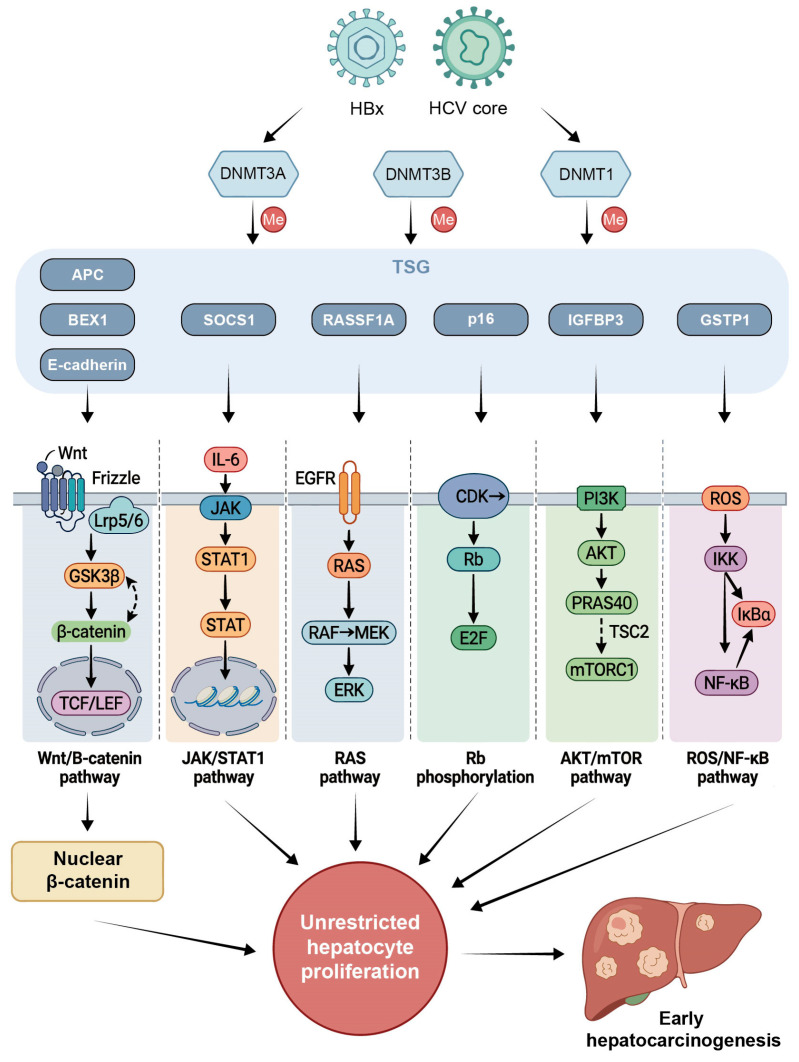
Schematic of canonical DNA methylation pathways in liver tumorigenesis, highlighting viral drivers and enzymatic writers. HBx and HCV infection promote aberrant CpG methylation through dysregulated DNMT activity, resulting in promoter hypermethylation and gene silencing, as well as global methylome shifts that contribute to oncogenic transformation. Created in BioRender. Zhou, L. (2026) https://BioRender.com/skgk3o1 (accessed on 2 July 2026).

Global DNA hypomethylation, alongside selective hypermethylation and silencing of TSG promoters containing CpG islands (e.g., *RASSF1A*, *P16*, *APC*, E-cadherin, *SOCS1*, *IGFBP3*, *GSTP1*, and *BEX1*), initiates at the liver preneoplastic/cirrhotic stage [38,39,40,41,42]. These hypermethylated TSGs are involved in some signaling pathways that induce hepatocarcinogenesis (Figure 2). For example, *RASSF1A*, a RAS inhibitor, inhibits cyclin D1 and hence induces G1 phase arrest. *RASSF1A* methylation might permit damaged hepatocytes to proceed further into the cell cycle by escaping G1 phase arrest [29]. Similarly, *SOCS1* is a JAK/STAT inhibitor. These hypermethylated inhibitors are concomitantly downregulated or lost and result in persistent activation of the RAS and JAK/STAT pathways in HCC [43]. The use of JAK/STAT and RAS inhibitors in combination with demethylating agents represents a therapeutic modality for HCC.

From a translational perspective, several methylation markers appear closest to clinical application. Promoter hypermethylation of classical tumor suppressors such as *RASSF1A*, *P16*, and *GSTP1* in circulating tumor DNA (ctDNA) has been consistently associated with HCC across independent cohorts, supporting their inclusion in blood-based detection panels [39,42]. More recently, methylation-sensitive high-resolution melting assays targeting *RNF135* and *LDHB*, particularly when combined with serum alpha-fetoprotein (AFP), have achieved encouraging sensitivity for early-stage HCC [44]. Genome-wide circulating cell-free DNA (cfDNA) 5-hydroxymethylcytosine signatures have also demonstrated high accuracy in distinguishing BCLC 0/A HCC from high-risk non-HCC populations [45]. Together, these data suggest that multi-marker methylation panels integrated with existing clinical scores may soon be ready for prospective validation in surveillance settings.

### 2.2. Histone Modifications

Nucleosomes are the basic structural units of chromatin. Each nucleosome consists of double-stranded DNA wrapped around a histone octamer formed by pairs of H2A, H2B, H3, and H4. The core histones contain flexible N-terminal tails, which can be modified by small chemical groups after protein synthesis. These changes are known as post-translational modifications (PTMs). By altering chromatin structure or recruiting specific proteins that recognize these marks, histone PTMs can regulate how tightly DNA is packaged and whether nearby genes are active or silent [46]. Histone modifications are reversible and dynamic. They are mainly controlled by “writer” and “eraser” enzymes, which add and remove PTM marks, respectively [47]. In addition, “reader” proteins, such as bromodomain-containing proteins (BRDs), recognize specific histone marks and help convert these epigenetic signals into downstream biological effects [48]. In this writer–eraser–reader framework, writers add chemical marks to histones or nucleic acids, erasers remove these marks, and readers interpret them. This concept helps explain why epigenetic regulators such as DNMTs, histone acetyltransferases (HATs), histone deacetylases (HDACs), histone methyltransferases (HMTases), and bromodomain and extra-terminal (BET) proteins are frequently investigated as therapeutic targets. The tails of histones H3 and H4 undergo several common PTMs, including phosphorylation, ubiquitination, acetylation, and methylation. These modifications influence chromatin accessibility and thereby affect gene transcription [32,49]. Emerging evidence has further implicated non-classical histone modifications, such as histone serotonylation and lactylation, in HCC [50,51].

#### 2.2.1. Histone Methylation

HMTases catalyze the methylation of histone residues, generating marks such as trimethylation of histone H3 lysine 27 (H3K27me3) and trimethylation of histone H3 lysine 4 (H3K4me3) [52]. Depending on the modified residue and methylation state, histone methylation may be associated with either compact, transcriptionally repressive chromatin or open, transcriptionally active chromatin. For example, H3K27me3 is generally associated with gene silencing, whereas H3K4me3 is considered a marker of active transcription [53]. In HCC, alterations in these methylation marks have been implicated in tumor progression. For example, H3K27me3 and H3K4me3 modifications at the *YTHDF2* promoter have been associated with poor prognosis, possibly through immune evasion and angiogenesis [54].

Enhancer of zeste homolog 2 (EZH2), the catalytic subunit of polycomb repressive complex 2 (PRC2), is a key H3K27 methyltransferase that mediates transcriptional repression of target genes in HCC [55]. EZH2 is frequently upregulated in HCC and appears to contribute to an aggressive tumor phenotype characterized by disease progression, vascular invasion, and increased cell proliferation [56]. Mechanistically, EZH2 colocalizes with H3K27me3 at promoter regions and directly silences TSGs, such as *CDKN2A*, *FOXO3*, *E2F1*, and *NOTCH2* [57]. By contrast, the menin-mixed lineage leukemia (MLL) HMTase complex is highly active in human HCC samples and drives hepatocarcinogenesis in a manner dependent on H3K4me3 [58]. Menin-mediated H3K4 methylation frequently occurs at proto-oncogene promoters, such as *PSMA7* and *CUL4A* [57]. Together, aberrant repressive and activating histone methylation programs contribute to HCC development.

In addition to H3K27me3 and H3K4me3, trimethylation of histone H3 lysine 9 (H3K9me3) is also associated with condensed and transcriptionally silent heterochromatin [59]. SET domain bifurcated histone lysine methyltransferase 1 (SETDB1), an H3K9-specific HMTase, is upregulated in HCC and is significantly associated with disease progression, tumor aggressiveness, and poor prognosis [60]. G9A, another HMTase, may also promote HCC progression by silencing TSGs such as *RARRES3* [61].

#### 2.2.2. Histone Acetylation

Histone acetylation is a widespread and reversible PTM regulated by two opposing enzyme classes: HATs, which act as “writers” by transferring acetyl groups from acetyl-CoA to lysine residues, and HDACs, which function as “erasers” by removing these acetyl groups [62].

Several HATs promote HCC progression by regulating glycolysis-related metabolic enzymes or cell cycle processes, including P300/CBP [63] and GCN5 [64]. In contrast, P300/CBP-associated factor (PCAF), another member of the HAT family, may act as a suppressor in HCC by promoting apoptosis through histone H4 acetylation and inhibition of AKT signaling [65]. Mechanistically, histone acetylation relaxes chromatin structure, thereby facilitating the binding of transcription factors to specific promoter regions and regulating multiple cellular signaling pathways [66]. For example, acetylation of the *IGF2BP3* promoter increases its expression and contributes to hepatocarcinogenesis by altering cyclin D1 messenger RNAs (mRNAs) stability, which subsequently affects cell cycle progression and cell proliferation [67].

BET family proteins act as “readers” of acetylated histones. By recognizing acetyl-lysine marks on chromatin, they help recruit transcriptional machinery and promote the expression of cancer-related genes such as *MYC* [68,69,70]. Among BET family members, BRD4 recognizes H3K27ac marks on chromatin through its bromodomains and regulates gene expression, while also participating in genomic integrity maintenance, DNA repair, cell death regulation, and telomere length control [71]. BRD4 is particularly enriched at super-enhancers, which are frequently associated with oncogene activation, and BRD4 occupancy is required for the transcription of these super-enhancer–driven oncogenes [72]. In HCC, c-MYC, a key regulator of cell proliferation and metabolism [73], is frequently overexpressed [74] and has been closely linked to tumorigenesis [75].

Human HDACs are commonly classified into four major classes based on their sequence homology to yeast HDACs, with class II further divided into class IIa and class IIb [76]. In HCC, HDACs regulate liver tumorigenesis, drug resistance, and metastasis by targeting specific gene promoters or interacting with key regulatory factors, thereby modulating downstream signaling pathways (Table 1). Although many HDACs have been implicated in HCC progression, some members may exert tumor-suppressive effects. For example, HDAC6 has been reported to suppress HCC by activating autophagic cell death through the JNK/BECLIN1 pathway [77]. In addition, certain HDACs may have context-dependent functions. Sirtuin 1 (SIRT1), for example, is often overexpressed in HCC and promotes tumorigenesis by maintaining telomere stability [78]. However, in the presence of mutant p53, SIRT1 may exert tumor-suppressive effects through the AMPK–mTOR pathway [79].

#### 2.2.3. Histone Serotonylation

Serotonin (5-hydroxytryptamine, 5-HT) is a neurotransmitter that has been implicated in tumorigenesis and tumor immunity by modulating innate and adaptive immune cells [96,97]. Recent evidence indicates that 5-HT also participates in liver carcinogenesis through histone serotonylation, a serotonin-derived epigenetic modification that links monoamine metabolism to chromatin regulation [98]. Histone serotonylation refers to the covalent attachment of serotonin to glutamine residues on histones. This modification was first identified as a transcription-related histone mark and has recently attracted attention in HCC because of its role in oncogenic transcription [50,98].

In HCC, nuclear-localized transglutaminase 2 (TGM2) catalyzes serotonylation of glutamine 5 on histone H3, generating H3Q5ser [98]. H3Q5ser acts as a permissive epigenetic mark that increases chromatin accessibility and transcriptional activity. Mechanistically, the TGM2–H3Q5ser axis promotes HCC progression mainly through *MYC* pathway activation. TGM2 expression is associated with higher AFP levels, poor tumor differentiation, and more advanced BCLC stage. In experimental models, *Tgm2* deficiency or H3Q5ser inhibition suppresses HCC progression. CUT&Tag and RNA-sequencing analyses further showed that genes downregulated after TGM2 inhibition were enriched in the *MYC* pathway [98]. TRIM28, also known as transcriptional intermediary factor 1β, may act as an adaptor protein that recruits TGM2 to the *MYC* promoter, thereby inducing H3Q5ser deposition and enhancing *MYC* transcription [50,98].

H3Q5ser also cooperates with classical active histone marks. TGM2-mediated H3Q5ser occurs adjacent to H3K4me3, a promoter-associated mark associated with active transcription [53]. Although H3Q5ser and H3K4me3 do not appear to directly regulate each other, their coexistence as H3K4me3Q5ser may amplify gene expression by enhancing transcriptional regulatory complex binding [50,99]. From a translational perspective, the TGM2–H3Q5ser axis may provide an indirect strategy to suppress MYC-driven HCC given that MYC itself remains difficult to target directly. In preclinical models, TGM2 inhibition suppressed HCC progression and showed synergistic activity with sorafenib, without obvious myelosuppression or tissue damage [98]. However, it remains to be clarified whether histone serotonylation reshapes MYC enhancer or super-enhancer activity, affects other histone modifications, or can be safely combined with kinase inhibitors or immunotherapy.

#### 2.2.4. Histone Lactylation

Lysine lactylation (Kla) is an emerging PTM in which lactyl groups are deposited on lysine residues, thereby linking cellular metabolism to epigenetic regulation [100]. In tumors, enhanced glycolysis, hypoxia, and lactate accumulation provide the metabolic basis for aberrant lactylation. Histone lactylation has been detected on H2A, H2B, H3, and H4, with H3K18la and H4K12la being among the best-characterized sites involved in transcriptional regulation [101]. Through this mechanism, lactate-derived lactyl groups influence chromatin-associated gene expression, allowing metabolic reprogramming to reshape transcriptional programs in cancer cells and the tumor microenvironment.

In HCC, Kla is not restricted to histones. Lactylation has also been identified in many non-histone proteins, suggesting that its regulatory functions extend beyond chromatin-associated transcriptional control. For example, lactylation of ABCF1 at lysine 430 is upregulated in HCC and promotes malignant progression by activating the HIF1 signaling pathway [102]. Global lactylome profiling in a prospective HBV-related HCC cohort detected 9275 Kla sites, including 9256 non-histone sites, indicating that lysine lactylation is a widespread modification in HCC [103].

Functionally, lactylation has been linked to multiple aspects of HCC progression, including tumorigenesis [102,103,104,105], metastasis [106,107], disease progression [108], and drug resistance [109]. Enhanced glycolysis, lactate accumulation, and lipid metabolic reprogramming may increase lactylation levels in HCC [103,105]. Lactylation-related regulation promotes HCC growth by activating oncogenic transcriptional programs. For example, lysine lactylation contributes to *AKR1B10* upregulation and alters the expression of downstream genes, including *LONP1*, *NPIPB3*, and *ZSWIM6*, thereby facilitating HCC cell proliferation [104]. In macrophages, histone lactylation-mediated *NUPR1* upregulation suppresses ERK and JNK signaling, creating an immunosuppressive milieu that supports HCC progression [107]. Moreover, H3K18la promotes *HECTD2* transcription, which facilitates KEAP1 degradation and activates the KEAP1/NRF2 antioxidant pathway, ultimately contributing to lenvatinib resistance in HCC cells [109].

From a translational perspective, histone lactylation provides a mechanistic link between the metabolic phenotype of HCC and epigenetic regulation. Lactate is no longer viewed only as a glycolytic byproduct; it can also serve as a metabolic fuel, signaling molecule, and substrate for lactylation, thereby shaping cancer cell behavior and the tumor microenvironment [110]. Lactylation-related marks or downstream gene signatures may serve as potential biomarkers for tumor progression, immune remodeling, and therapeutic response, especially in hypoxic or highly glycolytic tumors. In addition, lactylation-associated regulators, such as *HECTD2*, may represent candidate targets for overcoming treatment resistance. However, further studies are needed to identify the writers, readers, and erasers of lactylation in HCC and to determine whether targeting lactate metabolism or lactylation-associated pathways can improve current HCC therapies.

### 2.3. Noncoding RNAs

Much of the human genome is transcribed into RNA molecules without clear protein-coding potential [111]. These ncRNAs include intronic RNAs, microRNAs (miRNAs) [112], long noncoding RNAs (lncRNAs) [113], circular RNAs (circRNAs) [114], and extracellular RNAs [115]. The ENCODE project revealed that nonprotein-coding genomic regions generate thousands of RNA transcripts [116] that not only regulate fundamental biological processes, such as growth, development, and organ function, but also appear to play a critical role in the whole spectrum of human disease, notably cancer [117,118]. It is becoming clear that aberrant expression of ncRNAs is commonly observed across different types of cancer, suggesting that ncRNAs are key players in human carcinogenesis, including HCC [119,120]. Here, we focus on the roles of the two most studied classes of the ncRNA family, namely, miRNA and lncRNA.

#### 2.3.1. miRNA

miRNAs are endogenous, evolutionarily conserved, single-stranded ncRNAs approximately 20–24 nucleotides in length [121]. They regulate gene expression mainly after transcription by pairing with complementary sequences in the 3′-untranslated regions (3′-UTRs) of mRNAs, thereby influencing diverse biological processes [122]. Increasing evidence over the past decade has established miRNAs as important contributors to cancer initiation and progression [123].

**Figure 3:**
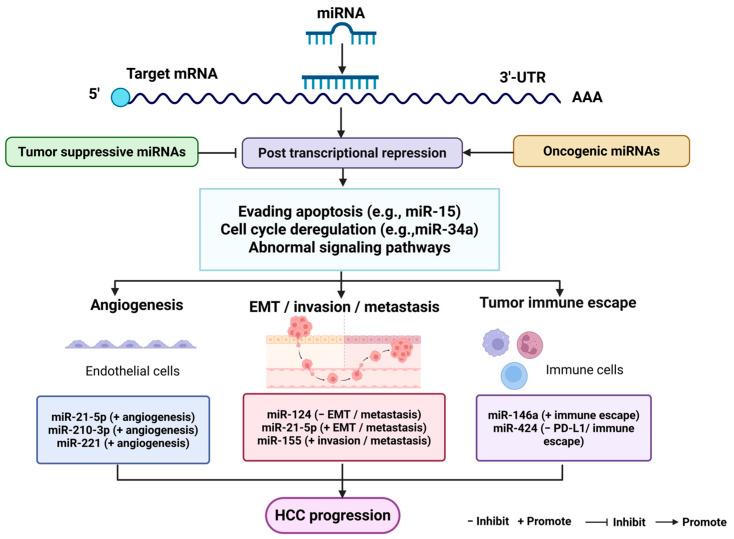
Roles of miRNAs in HCC progression. miRNAs regulate gene expression post-transcriptionally by binding to target mRNAs and inducing mRNA degradation or translational repression. In HCC, miRNAs function as oncogenic or tumor suppressive regulators depending on context. Oncogenic miRNAs (miR-21-5p [133], miR-210-3p [130], miR-221 [134]) promote angiogenesis, while tumor suppressive miRNAs inhibit malignant progression. miRNAs are involved in apoptosis, cell cycle regulation, and signaling pathways. miR-124 suppresses EMT and metastasis [135], whereas miR-21-5p [136] and miR-155 promote metastasis [137]. Tumor-derived miR-146a contributes to immune escape [132], while miR-424 negatively regulates immune checkpoint signaling [138]. Overall, miRNAs regulate angiogenesis, metastasis, immune escape, and HCC progression. Abbreviations: miRNA, microRNA; mRNA, messenger RNA; 3′-UTR, 3′-untranslated region; EMT, epithelial–mesenchymal transition; PD-L1, programmed death-ligand 1. Created in BioRender. Zhou, L. (2026) https://BioRender.com/5ldgozq (accessed on 1 July 2026).

In HCC, aberrant miRNA expression is frequently observed and is involved in the dysregulation of multiple cancer-related pathways, including mitochondrial apoptosis, cell cycle checkpoint control, proliferative signaling, and other regulatory networks [124]. Depending on their targets and biological context, miRNAs can function either as oncogenic factors or as tumor suppressors during HCC development (Figure 3). Their roles become particularly prominent during advanced diseases, in which metastasis is closely linked to EMT, angiogenesis, and increased invasive potential. For example, miR-124 restrains HCC invasion and metastasis by suppressing EMT through downregulation of the oncogenes *ROCK2* and *EZH2* [125].

Angiogenesis is one of the key processes influenced by miRNA-mediated regulation in HCC. Extracellular vesicles (EVs), which are endosome-derived vesicles of approximately 40–100 nm, are actively secreted by many cell types and transport functional cargos such as proteins, mRNAs, and miRNAs [126,127]. In HCC, tumor-derived EVs transfer miRNAs to cancer cells, stromal components of the tumor microenvironment, and distant organs, thereby supporting tumor growth, migration, and invasion [128,129]. These EV-associated miRNAs can also affect endothelial cells (ECs). For example, Lin et al. reported that HCC cells release exosomes carrying miR-210-3p, which enhances endothelial tube formation *in vitro*. Mechanistically, exosomal miR-210-3p promotes angiogenesis by repressing *SMAD4* and *STAT6* expression in ECs [130].

In addition to their roles in metastasis and angiogenesis, miRNAs are also involved in immune regulation. Tumor-derived miRNAs may facilitate immune escape by modulating immune checkpoint-related molecules [131]. In several cancers, reduced expression of certain miRNAs can relieve suppression of programmed death-ligand 1 (PD-L1) expression. In HCC, tumor-derived miR-146a induces M2 polarization of tumor-associated macrophages, which subsequently increases the expression of PD-1, TIGIT, and CTLA4 on T-cells and promotes HCC progression [132].

#### 2.3.2. lncRNAs

lncRNAs are nonprotein-coding transcripts longer than 200 nucleotides and serve as important regulators of gene expression [120]. More than 50,000 lncRNA-producing genes have been annotated to date, and this number continues to increase with advances in transcriptomic profiling [139]. Although lncRNAs are generally defined as noncoding transcripts, some can encode functional micropeptides that participate in HCC tumorigenesis [140]. A representative example is SMIM30, a micropeptide encoded by *LINC00998*. SMIM30 promotes HCC tumorigenesis by binding to SRC and YES1, enhancing their membrane anchoring and phosphorylation, and activating the downstream MAPK pathway [141].

Aberrant lncRNA expression is closely associated with HCC development and is controlled by regulatory mechanisms similar to those governing protein-coding genes, including epigenetic modifications, oncogenic or tumor-suppressive transcription factors, miRNAs, and RNA stability-related proteins [142]. Like miRNAs, lncRNAs may act either as oncogenic drivers or tumor suppressors depending on the biological context. During HCC progression, abnormal chromatin changes at lncRNA gene loci, including DNA methylation and histone modifications, suppress tumor-inhibitory lncRNAs or activate tumor-promoting lncRNAs [143]. For example, aberrant histone acetylation has been reported at the promoter regions of *H19*, *lncRNA-LET*, and *RUNX1-IT1* in HCC [144,145,146]. Under hypoxic conditions, HDAC3 acts as an upstream regulator that represses *RUNX1-IT1* expression, thereby enhancing proliferation and cancer stem-like properties in HCC cells [146]. In addition, DNMT1 and DNMT3 induce promoter hypermethylation of the tumor-suppressive lncRNA *MEG3*, leading to epigenetic silencing, apoptosis resistance, and increased liver tumor growth [147].

Many lncRNAs display tissue-specific expression patterns and are frequently dysregulated in cancers, including HCC [148]. Functionally, lncRNAs participate in HCC progression through multiple mechanisms, such as acting as miRNA sponges, regulating mRNA stability, interacting with regulatory proteins, and encoding functional micropeptides (Table 2).

Some lncRNAs influence tumor-related signaling by interacting with key regulatory proteins. For example, lncRNA *SNHG5* directly binds to UPF1 and activates the Wnt/β-catenin pathway, thereby maintaining cancer stem cell properties and promoting tumor proliferation in HCC [149].

lncRNAs also regulate HCC progression by modulating RNA stability. For example, lncRNA *FTO-IT1* is upregulated in HCC and stabilizes FTO mRNA by recruiting the ILF2/ILF3 protein complex to its 3′-UTR. Increased FTO expression then alters m6A modification of glycolysis-related genes, including *GLUT1*, PKM2, and c-MYC, thereby enhancing glycolytic metabolism and promoting HCC progression [158].

Another well-characterized mechanism is the competitive endogenous RNA (ceRNA) model, in which lncRNAs bind miRNAs and function as miRNA sponges. In HCC, some lncRNA-mediated ceRNA networks exert tumor-suppressive effects. For example, lncRNA *XIST* suppresses HCC proliferation and metastasis by interacting with miR-92b [159]. In contrast, other lncRNAs may facilitate malignant progression or treatment resistance. For example, lncRNA *TTN-AS1* enhances sorafenib resistance in HCC by sponging miR-16-5p and subsequently upregulating cyclin E1 [160].

### 2.4. Crosstalk Between Epigenetic Layers

Epigenetic alterations in HCC do not act independently. Instead, DNA methylation, histone modifications, and noncoding RNAs can interact with each other and jointly regulate gene expression. This crosstalk may strengthen oncogenic signaling or stabilize TSG silencing during HCC development.

lncRNAs can regulate epigenetic enzymes and participate in feedback loops. For example, *HOTAIR* suppresses miR-122 expression through DNMT-mediated DNA methylation and further increases DNMT expression via EZH2. The repression of miR-122 reactivates the oncogene Cyclin G1, thereby enhancing the tumorigenic potential of HCC cells [161].

miRNA expression can also be controlled by epigenetic mechanisms. EZH2, a key H3K27 methyltransferase, epigenetically silences several tumor-suppressive miRNAs in HCC, including miR-139-5p [162], miR-125b [163], miR-101 [164], let-7c [165], thereby contributing to metastatic progression [166]. Conversely, some tumor-suppressive miRNAs can target epigenetic modifiers. For example, miR-26a inhibits EMT by downregulating EZH2 and restoring E-cadherin expression [167].

DNA methylation and histone modifications can also cooperate to maintain transcriptional repression. Methylated promoters can recruit methyl-CpG-binding proteins, which further attract HDACs and histone methyltransferases, resulting in compact chromatin. Conversely, repressive histone marks such as H3K9 methylation can help recruit DNMTs and maintain DNA methylation, forming a stable gene-silencing loop [168]. In HCC, hypermethylation of the *3OST2* promoter is associated with reduced *3OST2* expression. UHRF1 and the repressive mark H3R8me2s are enriched at this promoter. Treatment with 5-Aza-CdR, a DNA methylation inhibitor, or TSA, an HDAC inhibitor, decreases UHRF1 and H3R8me2s enrichment and restores *3OST2* expression [169].

Together, these findings suggest that epigenetic layers in HCC are interconnected rather than isolated, and their crosstalk may amplify malignant phenotypes.

## 3. Epigenetic Biomarkers of Molecular Diagnosis in HCC

The poor survival of HCC patients is mainly due to delayed diagnosis and limited therapeutic efficacy in advanced disease. Early-stage HCC is frequently asymptomatic, making timely detection difficult, whereas advanced or metastatic HCC often has limited treatment options and poor clinical outcomes [170]. These challenges underscore the need for reliable biomarkers that can improve surveillance, enable early detection, and identify premalignant or early malignant lesions.

At present, HCC surveillance primarily depends on ultrasonography and serum AFP, occasionally supplemented by additional imaging examinations. Tissue biopsy remains useful when diagnosis or treatment planning requires histological confirmation, but its invasive nature and limited ability to represent tumor heterogeneity restrict its broader application. Liquid biopsy refers to the detection of tumor-derived molecules in body fluids, most commonly blood. Compared with tissue biopsy, liquid biopsy can be repeated over time and may better capture dynamic tumor changes [171]. Although blood is the most commonly used specimen, urine, ascites, bile, saliva, and other body fluids may also be analyzed [172]. Liquid biopsy biomarkers include AFP and other serum markers, circulating tumor cells, tumor-derived EVs, circulating tumor DNA, aberrant ctDNA methylation, and ncRNAs [173]. Owing to its repeatability and ability to reflect tumor heterogeneity, liquid biopsy is increasingly regarded as a promising tool for HCC management [173].

### 3.1. DNA Methylation-Related Biomarkers in HCC

Aberrant DNA methylation, especially promoter hypermethylation of TSGs and hypomethylation of oncogenes, is an important mechanism of cancer-related gene dysregulation. With advances in molecular detection technologies, gene-specific methylation alterations have increasingly been explored as biomarkers for cancer diagnosis and monitoring [172,174]. Because DNA methylation changes may occur during early tumor initiation and can precede detectable somatic mutations, they provide an opportunity for cancer detection before clinical symptoms appear. In HCC, methylation-based assays have shown diagnostic potential and may help reduce false-negative results compared with mutation-centered approaches [175].

Among the reported methylation biomarkers, *RASSF1A* promoter hypermethylation is one of the most extensively studied in HCC. RASSF1A functions as a tumor suppressor and inhibits tumorigenesis by inducing cell cycle arrest, limiting cell migration, stabilizing microtubules, and promoting apoptosis [176,177,178]. Circulating methylated *RASSF1A* has shown diagnostic value in HCC, with a detection rate of 93% and an AUC of 0.81 for distinguishing HCC patients from HBV carriers. When combined with AFP, methylated *RASSF1A* improved diagnostic performance compared with AFP alone, with 77% sensitivity and 89% specificity versus 65% and 87%, respectively [179]. However, *RASSF1A* remains an early single-gene marker, and its clinical utility requires broader external validation.

Liquid biopsy further expands the clinical applicability of methylation-based biomarkers. ctDNA, a tumor-derived fraction of cfDNA, carries molecular information released from tumor cells into the bloodstream [180]. Methylated ctDNA and cfDNA hydroxymethylation profiles are, therefore, promising sources of plasma biomarkers for early cancer detection, prognostic assessment, and disease surveillance [181]. With next-generation sequencing, bisulfite sequencing, and PCR-based assays, circulating DNA can be analyzed for tumor-specific mutations, methylation changes, and other molecular abnormalities [182].

Several circulating DNA methylation and hydroxymethylation signatures have shown diagnostic value in HCC. In a large cfDNA 5hmC-Seal study including 2554 Chinese subjects, a 32-gene 5-hydroxymethylcytosine model distinguished BCLC stage 0/A HCC from non-HCC controls with an AUC of 0.884 in the validation set and differentiated early HCC from high-risk subjects with CHB or cirrhosis with an AUC of 0.846 [45]. More recently, a multicenter cohort study showed that plasma cfDNA methylation outperformed mutation-based ctDNA analysis for HCC detection. A 25-marker methylation assay achieved an AUC of 0.958, with 86.7% sensitivity and 90.1% specificity, whereas a simplified two-marker qMSP assay, HCCtect, based on *OTX1* and *HIST1H3G* achieved an AUC of 0.925, with 78.4% sensitivity and 93.0% specificity. HCCtect also detected early-stage HCC with an AUC of 0.896, 69.5% sensitivity, and 93.0% specificity [183]. These data support cfDNA methylation-based profiling as a promising and clinically accessible approach for non-invasive HCC detection. However, broader external validation in international populations, non-HBV-related etiologies, and prospective longitudinal screening cohorts is still needed before routine clinical implementation.

### 3.2. miRNAs as Biomarkers in HCC

Altered circulating miRNA profiles have attracted increasing attention as non-invasive biomarkers for HCC diagnosis and prognosis. Compared with conventional serum markers such as AFP, circulating miRNAs are relatively stable in body fluids and can reflect tumor-associated molecular alterations. Their stability is partly attributed to their resistance to RNase activity, extreme pH, and temperature changes [184,185].

Several circulating miRNAs have shown diagnostic potential in HCC. A recent meta-analysis including 36 studies, 3362 patients with HCC, and 2150 patients with chronic hepatitis showed that circulating miRNAs had a pooled sensitivity of 0.79, specificity of 0.79, and SROC AUC of 0.85 for HCC diagnosis [186]. In the validation analysis, miR-1246, miR-21, and miR-221 were significantly upregulated in HCC patients compared with patients with chronic hepatitis (*p* < 0.01), whereas miR-122 and miR-26a were downregulated (*p* < 0.01) [186].

Earlier meta-analytic evidence further showed that miR-21 and miR-122 individually distinguished HCC from healthy controls, with AUCs of 0.88 and 0.77, respectively; the corresponding sensitivity and specificity were 86.6% and 79.5% for miR-21 and 68.0% and 73.3% for miR-122 [187].

Compared with single miRNAs, multi-miRNA panels may provide more reliable diagnostic performance. In a large study including 934 participants with independent training and validation cohorts, a plasma seven-miRNA panel composed of miR-122, miR-192, miR-21, miR-223, miR-26a, miR-27a, and miR-801 achieved AUCs of 0.864 and 0.888 in the training and validation datasets, respectively [188]. In the validation dataset, this panel showed 81.8% sensitivity and 83.5% specificity for HCC diagnosis. It also distinguished HCC from healthy controls and patients with CHB and cirrhosis with AUCs of 0.941, 0.842, and 0.884, respectively [188].

Although these findings support circulating miRNAs as promising liquid biopsy biomarkers, miRNA-based assays have not entered routine clinical practice to date. Their clinical translation remains limited by differences in sample type, RNA extraction, normalization strategies, detection platforms, and insufficient prospective validation across different etiologies.

### 3.3. lncRNAs as Biomarkers in HCC

Several lncRNAs related to HCC are detectable in body fluids, making them suitable candidates for liquid biopsy-based assessment. Their expression profiles often differ between HCC tissues and normal liver tissues or non-HCC controls, suggesting potential value in tumor detection and patient stratification [189]. Circulating lncRNAs are, therefore, being increasingly explored as minimally invasive tools for early diagnosis, disease surveillance, and outcome prediction [190].

However, the diagnostic performance of single circulating lncRNAs remains variable. Some reported candidates, such as *lncRNA-WRAP53*, *LINC01225*, and *HULC*, have been associated with HCC diagnosis, recurrence, or prognosis [191,192,193]. Nevertheless, many of these markers were evaluated in relatively small cohorts, and complete diagnostic indices, including sensitivity, specificity, and AUC, are not consistently available. In addition, some HCC-associated lncRNAs may also be altered in other cancer types [194] or in non-malignant liver disorders, including cirrhosis and liver injury [195], which limits their disease specificity.

Combining lncRNAs with established clinical markers may improve diagnostic performance. For example, a serum exosomal panel composed of *ENSG00000258332.1*, *LINC00635*, and AFP achieved an AUC of 0.894, with 83.6% sensitivity and 87.7% specificity, for distinguishing HCC from CHB; this result was further confirmed in an independent validation cohort [196]. Similarly, the combination of *UCA1*, *WRAP53*, and AFP increases diagnostic sensitivity to 100%, although complete diagnostic indices such as AUC and specificity were not consistently reported [191]. These findings suggest that lncRNA-based panels may have greater clinical potential than single lncRNA markers.

Overall, circulating lncRNAs represent promising liquid biopsy biomarkers for HCC, particularly when used in combination with AFP or other molecular markers. Nevertheless, compared with cfDNA methylation and miRNA panels, current evidence for circulating lncRNAs remains less mature. Further validation in large, multicenter cohorts and careful evaluation of specificity across different liver diseases are still required before routine clinical implementation.

### 3.4. Extracellular Vesicle-Associated ncRNAs as Liquid Biopsy Biomarkers in HCC

EVs have become an important source of liquid biopsy biomarkers in HCC. EVs are lipid bilayer-enclosed nanoscale particles released by most mammalian cells into the extracellular space, where they mediate local and distant intercellular communication. According to their origin and size, EVs are generally classified into exosomes, microvesicles, and apoptotic vesicles. Exosomes are small EVs of approximately 30–150 nm and are generated through the endosomal multivesicular body pathway. In contrast, microvesicles are produced by outward budding of the plasma membrane, whereas apoptotic vesicles are released during programmed cell death [197,198]. EVs contain diverse molecular cargos, including DNA, mRNAs, miRNAs, lncRNAs, circRNAs, proteins, lipids, and metabolites. Because these cargos can partly mirror the molecular features of their parental cells, tumor-derived EVs may provide a stable and minimally invasive source of information for HCC detection and surveillance [197].

The diagnostic value of EVs is closely connected with their ncRNA cargo. Encapsulation within EVs can protect miRNAs, lncRNAs, and circRNAs from degradation in the circulation, which improves their stability and detectability in liquid biopsy [198]. Among these cargos, exosomal miRNAs have been widely investigated because of their abundance, stability, and accessibility in body fluids. In HCC, a serum exosome-derived three-miRNA signature composed of miR-122-5p, let-7d-5p, and miR-425-5p showed promising diagnostic performance, with AUCs of 0.905 and 0.954 in the training and validation sets, respectively. This signature also detected low-stage HCC with an AUC of 0.923 [199]. EV-associated lncRNAs have also been explored as diagnostic candidates, as some lncRNAs show different expression patterns between HCC-derived exosomes and non-malignant controls. For example, serum exosomal *LINC00161* has been reported as an exosome-associated lncRNA biomarker for HCC, with an AUC of 0.794 in a training and validation study [200]. In addition, EV-associated circRNAs have been evaluated as potential diagnostic and prognostic biomarkers in HCC [198]. However, EV-based biomarkers are still not ready for routine clinical use because EV isolation, detection platforms, tumor-specific EV enrichment, and prospective clinical validation remain insufficiently standardized [197].

## 4. Preclinical and Clinical Translation of Epigenetic Therapies in HCC

**Figure 4:**
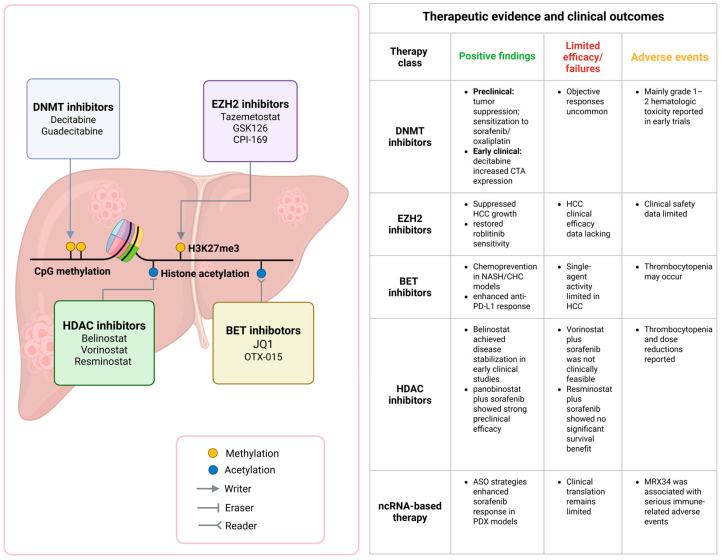
Epigenetic therapeutic strategies and current evidence in HCC. Representative epigenetic therapies target DNA methylation, histone acetylation, histone methylation, and reader-mediated chromatin regulation. DNMT inhibitors, HDAC inhibitors, EZH2 inhibitors, BET inhibitors, and ncRNA-based therapies have shown tumor-suppressive or treatment-sensitizing effects in preclinical HCC models. However, clinical translation remains limited by modest single-agent activity, insufficient HCC-specific efficacy data, small early-phase trials, and treatment-related adverse events, including hematologic toxicity, thrombocytopenia, and immune-related toxicities. Abbreviations: DNMT, DNA methyltransferase; HDAC, histone deacetylase; EZH2, enhancer of zeste homolog 2; BET, bromodomain and extra-terminal domain; ncRNA, noncoding RNA; CTA, cancer-testis antigen; PDX, patient-derived xenograft; NASH, nonalcoholic steatohepatitis; CHC, chronic hepatitis C; PD-L1, programmed death-ligand 1; ASO, antisense oligonucleotide. Created in BioRender. Zhou, L. (2026) https://BioRender.com/dgyhz1h (accessed on 2 July 2026).

Epigenetic dysregulation contributes to HCC initiation, progression, immune escape, and therapeutic resistance, making epigenetic regulators attractive targets for therapeutic intervention. Epigenetic drugs evaluated in HCC mainly include inhibitors of DNMTs, histone-modifying enzymes, chromatin readers, and ncRNA-directed therapeutic strategies. Preclinical studies have shown that these approaches can suppress HCC cell proliferation, promote apoptosis or ferroptosis, reverse oncogenic transcriptional programs, and enhance sensitivity to targeted therapy or immune checkpoint blockade. However, their clinical translation remains challenging. Although early clinical trials of DNMT inhibitors, HDAC inhibitors, and miRNA-based therapeutics have provided preliminary evidence of tolerability, disease stabilization, or pharmacodynamic activity, objective responses and survival benefits have generally been limited, and some combinations have been restricted by treatment-related toxicity. Therefore, current evidence supports epigenetic therapy more strongly as a rational combination or sensitizing strategy than as monotherapy in HCC. In this section, we summarize the evidence according to experimental level, including cell-based studies, animal and xenograft models, early clinical studies, and preclinical combination strategies (Figure 4).

### 4.1. Cell-Based Evidence for Epigenetic Therapy

Cell-based studies have provided mechanistic evidence supporting epigenetic therapy in HCC. One strategy is to restore tumor-suppressive miRNAs that normally restrain oncogenic epigenetic regulators [201]. For example, enforced miR-26a expression in HepG2 cells decreased EZH2 expression, induced G1-phase cell-cycle arrest, and inhibited cell proliferation, suggesting that miRNA replacement may indirectly suppress EZH2-dependent oncogenic activity [202].

In addition to miRNAs, lncRNA-directed approaches have also been explored in HCC cell models. RNAi, CRISPR interference, and antisense oligonucleotides provide useful tools for reducing disease-promoting lncRNAs or suppressing their transcription [203,204]. A pooled shRNA-based screen identified *ASTILCS* as an essential regulator of HCC cell survival. *ASTILCS* was overexpressed in HCC tumors, and its functional importance was validated using RNAi, CRISPRi, and ASOs. Knockdown of *ASTILCS* induced apoptosis and was associated with downregulation of the neighboring survival-related gene *PTK2*, suggesting that *ASTILCS* may represent a potential lncRNA-based therapeutic target in HCC [205].

### 4.2. Animal and Xenograft Evidence

Animal and xenograft models have provided important *in vivo* evidence supporting epigenetic therapy in HCC. For EZH2-targeted therapy, AR-driven activation of EZH2-mediated Wnt/β-catenin signaling promoted hepatocarcinogenesis, and this axis was validated using xenograft and orthotopic mouse models. These findings suggest that EZH2 may function as a key epigenetic mediator linking hormone-related signaling to HCC progression [206].

BET/BRD4 inhibition has also shown therapeutic potential in animal models of liver cancer. In nonalcoholic steatohepatitis (NASH)- and chronic hepatitis C (CHC)-associated liver disease models, pharmacological inhibition of BRD4 suppressed liver disease progression and hepatocarcinogenesis by reversing cancer risk–associated transcriptional programs [207]. In fibrotic orthotopic HCC mouse models, the BET bromodomain inhibitor i-BET762 enhanced the efficacy of anti-PD-L1 therapy by suppressing monocytic myeloid-derived suppressor cells, increasing tumor-infiltrating lymphocytes, promoting tumor eradication, and prolonging survival [208]. Another HCC mouse study showed that JQ1 alone had limited antitumor efficacy but upregulated PD-L1 expression; importantly, JQ1 combined with PD-L1 blockade enhanced CD8^+^ T-cell-mediated antitumor responses and suppressed HCC progression *in vivo* [209].

HDAC inhibitors have also been evaluated in HCC animal models. Panobinostat, a pan-HDAC inhibitor, inhibited HCC growth and metastasis *in vitro* and *in vivo* partly through suppression of the gankyrin/STAT3/Akt pathway [210]. In another HCC-specific preclinical study, panobinostat showed marked antitumor activity in liver cancer cell lines and xenograft models, and its combination with sorafenib further reduced vessel density and tumor volume while improving survival [211]. These findings support the potential of HDAC inhibition, particularly in combination with targeted therapy, as a preclinical strategy for HCC treatment.

### 4.3. Early Clinical Studies and Therapeutic Outcomes

Early clinical studies have evaluated several epigenetic therapies in patients with HCC or advanced solid tumors involving the liver. For DNMT inhibition, hepatic arterial infusion of decitabine was tested in a phase I trial in patients with unresectable liver-predominant metastases from solid tumors. The regimen was feasible and tolerable, with mainly grade 1–2 hematological toxicities and no dose-limiting toxicity. Although no objective tumor responses were observed, post-treatment biopsies showed increased expression of cancer-testis antigens, suggesting enhanced tumor immunogenicity and providing a rationale for future combinations with immune checkpoint blockade [212].

HDAC inhibitors have also entered early clinical evaluation in HCC. In a multicenter phase I/II study of belinostat in patients with unresectable HCC and CLD, the maximum tolerated dose was not reached, and the treatment was generally well tolerated. However, the objective response rate was low, with a partial response rate of 2.4% and stable disease rate of 45.2%. Median progression-free survival and overall survival were 2.64 and 6.60 months, respectively. Exploratory analysis suggested that high HR23B expression may be associated with disease stabilization [213].

Several HDAC inhibitor-based combinations have been tested with sorafenib. A phase I trial of vorinostat plus sorafenib in unresectable HCC showed durable disease stabilization in some patients, but frequent toxicities and dose modifications prevented determination of a recommended phase II dose, limiting further development of this schedule [214]. Similarly, a randomized phase I/II trial comparing resminostat plus sorafenib with sorafenib alone in advanced HCC did not show significant improvement in time-to-progression or overall survival. Grade ≥3 thrombocytopenia was more frequent in the combination group, highlighting the toxicity concerns of HDAC inhibitor-based combinations [215].

For ncRNA-based therapy, MRX34, a liposomal miR-34a mimic, was evaluated in a first-in-human phase I trial in patients with advanced solid tumors, including HCC [216]. Pharmacodynamic analyses confirmed miR-34a delivery and dose-dependent modulation of target genes [216]. Although partial responses and durable stable disease were observed in a subset of patients, the trial was terminated early because of serious immune-mediated adverse events, including four patient deaths. This study provided clinical proof-of-concept for miRNA replacement therapy but also underscored major safety barriers to systemic miRNA therapeutics [216].

Overall, early clinical studies suggest that epigenetic therapies in HCC have shown manageable toxicity in selected settings and occasional disease stabilization, but objective responses and survival benefits remain limited. Combination strategies with targeted therapy, chemotherapy, or immunotherapy may improve antitumor activity, but treatment-related toxicity and lack of clear predictive biomarkers remain major obstacles to clinical translation.

In contrast, clinical evidence for EZH2 and BET inhibitors in HCC remains limited. Although EZH2 inhibitors such as tazemetostat and several BET inhibitors have entered early-phase clinical trials in hematological malignancies and selected solid tumors, HCC-specific clinical efficacy data are still lacking. Therefore, current support for EZH2- and BET-targeted therapy in HCC mainly comes from preclinical studies, including *in vitro* assays, xenograft models, chemically induced or diet-induced HCC models, and combination immunotherapy models.

### 4.4. Epigenetic Drugs as Sensitizing Agents in Preclinical Models

Epigenetic drugs have increasingly been investigated as sensitizing agents to improve the efficacy of targeted therapy, chemotherapy, and immunotherapy in HCC. Because epigenetic repression can maintain drug-resistant transcriptional programs, combined targeting of different epigenetic layers may produce stronger antitumor effects than single-agent treatment. For example, dual inhibition of DNA methylation and EZH2-mediated histone repression using 5-aza-2-deoxycytidine and GSK126 enhanced nucleosome accessibility, reactivated genes that remained silenced after DNMT inhibition alone, and produced sustained anti-proliferative effects in HCC cell models [217].

Epigenetic inhibition may also restore sensitivity to targeted molecular therapy. FGFR4 blockade with roblitinib was shown to induce non-canonical NF-κB-dependent EZH2 accumulation, which contributed to treatment resistance. EZH2 knockdown or pharmacological inhibition with CPI-169 sensitized HCC cells to roblitinib, while the combination of roblitinib and CPI-169 synergistically induced apoptosis and suppressed tumor growth in zebrafish and mouse HCC models [218]. Similarly, lncRNA-targeted strategies may enhance tyrosine kinase inhibitor sensitivity. ASO-mediated inhibition of the hypoxia-induced lncRNA *SHIELD* promoted ferroptosis through the GRSF1-GPX4 axis and potentiated the antitumor activity of sorafenib in HCC patient-derived xenograft models [219]. Loss of *LINC01056*, by contrast, reduced sorafenib sensitivity through PPARα-dependent metabolic reprogramming toward fatty acid oxidation, and inhibition of this pathway restored sorafenib responsiveness *in vitro* and *in vivo* [220].

HDAC inhibitors have also been explored as sensitizers to targeted therapy. Panobinostat enhanced the antitumor activity of sorafenib in HCC preclinical models, reducing cell viability and proliferation while increasing apoptosis and autophagy. In xenografts, the combination decreased vessel density and tumor volume and improved survival [211]. Entinostat also enhanced the cytotoxic effect of lenvatinib in liver cancer cells by inducing ROS-dependent ATM activation, modulating mTORC1/2 and eIF2α signaling, promoting autophagy, and reducing anti-apoptotic proteins such as MCL-1 and BCL-XL [221].

In addition to targeted therapy, epigenetic drugs may sensitize HCC to immunotherapy by reshaping the tumor immune microenvironment. In fibrotic HCC models, the BET inhibitor i-BET762 suppressed monocytic myeloid-derived suppressor cells and enhanced anti-PD-L1 efficacy, leading to increased tumor-infiltrating lymphocytes, tumor eradication, and prolonged survival [208]. Another study showed that JQ1 alone had limited activity in HCC but increased PD-L1 expression; when combined with PD-L1 blockade, it enhanced CD8^+^ T-cell-mediated antitumor responses and suppressed HCC progression *in vivo* [209]. These findings suggest that BET inhibition may be more effective as an immunotherapy-sensitizing strategy than as monotherapy in HCC.

Overall, these preclinical studies indicate that epigenetic agents can act as sensitizers by reversing drug-resistant chromatin states, restoring silenced therapeutic targets, promoting ferroptosis or autophagy, and enhancing antitumor immunity. However, most of these strategies remain at the cell or animal-model stage, and their clinical benefit in HCC still requires validation in well-designed trials.

## 5. Challenges in Clinical Translation of Epigenetic Therapies

Although epigenetic therapies have shown strong mechanistic rationale in HCC models, their clinical benefits in HCC and other solid tumors remain limited. DNMT inhibitors, HDAC inhibitors, BET inhibitors, EZH2 inhibitors, and ncRNA-based therapeutics can suppress tumor growth, reverse drug resistance, or enhance immune recognition in preclinical studies [212,216,218,222,223,224,225,226,227,228,229,230,231,232]. However, these effects have not consistently translated into improved outcomes in patients with HCC. Possible reasons include tumor heterogeneity, insufficient drug penetration into solid tumors, unfavorable pharmacokinetic properties such as short half-lives for some compounds, dynamic and reversible epigenetic states, limited single-agent activity, and a lack of biomarker-guided patient selection [225,226,232,233].

Clinical trial design is another important limitation. Most HCC-related studies of epigenetic drugs have been early-phase trials with small sample sizes and limited statistical power [212,213,214,215,217,234,235]. Their main endpoints were often safety, tolerability, recommended dose, or pharmacodynamic activity, rather than objective response rate, progression-free survival, or overall survival. In addition, many trials did not select patients according to DNMT, HDAC, EZH2, or BRD4 expression; DNA methylation signatures; histone modification profiles; immune contexture; or ncRNA expression patterns. This may dilute potential benefits in molecularly responsive subgroups.

Toxicity further limits clinical translation. DNMT inhibitors may cause hematologic toxicity, infection risk, and hepatic dysfunction, and SGI-110 may raise safety concerns when used as long-term repeated treatment [222,223]. HDAC inhibitors are associated with fatigue, gastrointestinal symptoms, myelosuppression, and hepatic toxicity. For BET inhibitors, thrombocytopenia, gastrointestinal toxicity, fatigue, and adaptive resistance have been reported or proposed as potential concerns based on broader oncology studies [225,226,232]. For EZH2 inhibition, HCC heterogeneity may lead to variable treatment responses, and acquired resistance may limit durable benefit [224]. These risks are particularly relevant in patients with cirrhosis or impaired liver reserve.

The MRX34 trial highlights the challenges of ncRNA-based therapy. MRX34, a liposomal miR-34a mimic, showed preliminary activity in advanced solid tumors, including HCC. However, the trial was terminated early because of serious adverse events, including treatment-related deaths [216]. This failure may reflect the broad target spectrum of miRNA mimics, immune activation caused by systemic liposomal delivery, cytokine release, off-target gene regulation, and a narrow therapeutic window.

Future studies should, therefore, prioritize biomarker-guided patient selection, optimization of dose and treatment interval, improved drug stability and pharmacokinetic exposure, tumor-targeted delivery systems, rational combination strategies, pharmacodynamic endpoints, and multicenter randomized trials with sufficient statistical power. For HCC specifically, liver function stratification, cirrhosis status, and treatment-related hepatic toxicity should be incorporated into trial design.

## 6. Conclusions and Future Perspectives

Epigenetic dysregulation is involved in HCC initiation, progression, immune escape, therapeutic resistance, and biomarker development. DNA methylation, histone modifications, chromatin remodeling, ncRNAs, and EV-associated ncRNAs provide potential opportunities for diagnosis and therapy. However, these mechanisms should not be considered equally mature for clinical translation.

Among current epigenetic biomarkers, DNA methylation and hydroxymethylation signatures have relatively stronger clinical evidence, especially because several cfDNA-based panels have been evaluated in large cohorts with reported sensitivity, specificity, and AUC. In contrast, many miRNA, lncRNA, and EV-associated ncRNA biomarkers remain more exploratory. Their diagnostic or prognostic performance may vary across studies because of differences in HCC etiology, tumor stage, liver disease background, specimen type, detection platform, normalization method, and cohort design. Similar caution is needed for epigenetic therapies given that DNMT inhibitors, HDAC inhibitors, EZH2 inhibitors, BET inhibitors, and ncRNA-based therapies have shown preclinical activity but limited clinical benefit in HCC.

Future research should move from single-marker analysis toward integrative and context-specific approaches. Multi-omics integration may help clarify how genomics, epigenomics, transcriptomics, proteomics, metabolomics, and microbiome profiles cooperate during hepatocarcinogenesis [236]. Spatial epigenomics and single-cell technologies may reveal cell type-specific epigenetic states within the HCC microenvironment [237]. Artificial intelligence (AI)-driven biomarker discovery may further integrate molecular, imaging, pathological, and clinical data to identify robust biomarker panels and predict treatment response [238]. Etiology-specific strategies are also needed because HBV-related, HCV-related, alcohol-associated, and MASLD-related HCC may have distinct epigenetic landscapes.

Overall, epigenetic research has expanded our understanding of HCC biology, but successful clinical translation will require standardized detection methods, prospective multicenter validation, biomarker-guided patient selection, careful toxicity evaluation, and rational combination strategies. These efforts may help transform epigenetic discoveries into clinically useful tools for precision management of HCC.

## Figures and Tables

**Table 1:** Classification of HDACs and their functions in HCC.

Class	Name	Expression	Interaction Factors	Target Histone	Target Molecules	Pathways	Results	Ref.
I	HDAC1	Up	HIF1-α	H3	*FAM99A*	Hypoxia signal transduction, EMT (+)	Metastasis (+)	[80]
	HDAC2	Up	N/A	H3K9, H3K27	LAPTM4B	Autophagy pathway	Proliferation (+)	[81]
	HDAC3	Down	H3K9ac/H3K9me3	H3K9	KRAS, CDK6, ERCC1	DNA damage repair pathway	DNA repair defect, tumorigenesis	[82]
	HDAC8	Up	T-cell-trafficking chemokines	H3K27	CCL4	CD8^+^ T-cell immune microenvironmental pathway	ICIs resistance	[83]
IIa	HDAC4	Up	MEF2D	N/A	SPRY4	RAS/MAPK/ERK pathway	Kinase inhibitor resistance	[84]
	HDAC5	Up	TBX3	N/A	E-cadherin	EMT (+)	Migration (+), metastasis (+)	[85]
	HDAC7	Up	PU.1	H3K89	ENO1	Cell cycle pathway	Proliferation (+)	[86]
	HDAC9	Up	ALDH	N/A	ALDH1A3	CSCs pathway	Proliferation (+), metastasis (+)	[87]
IIb	HDAC6	Up	JNK/c-Jun activation	N/A	Beclin-1	Beclin 1-dependent autophagic cell death pathway	Proliferation (−)	[77]
	HDAC10	Up	miR-3178	N/A	miR-3178	AKT pathway	Proliferation (+), apoptosis (−)	[88]
III	SIRT1	Up	TERT, PTOP	N/A	N/A	Telomere maintenance	Proliferation (+), apoptosis (−)	[78]
	SIRT2	Up	E/CDK, p300	N/A	ANXA2	mTOR pathway	Kinase inhibitor resistance	[89]
	SIRT3	Down	N/A	N/A	CCNE2	Cell cycle pathway	Proliferation (−), metastasis (−), apoptosis (+)	[90]
	SIRT4	Up	CaMKII γ	H3K27	MCCC2	WNT/β-catenin and AKT pathways, EMT (+)	Migration (+), metastasis (+)	[91]
	SIRT5	Down	FXRα(*NR1H4*), TCA	N/A	ACOX2	Immunosuppressive microenvironmental pathway	Proliferation (+)	[92]
	SIRT6	Up	N/A	H3K9	BAX	BCL2–BAX protein-dependent apoptotic pathway	Proliferation (+)	[93]
	SIRT7	Up	N/A	N/A	p53	p53-NOXA pathway	TACE resistance	[94]
IV	HDAC11	Up	N/A	N/A	LKB1	AMPK and the glycolysis signaling pathway	Proliferation (+), kinase inhibitor resistance	[95]

EMT, epithelial–mesenchymal transition; TCA, taurocholic acid; CSCs, cancer stem cells; CCNE2, Cyclin E2; TACE, transarterial chemoembolization; N/A, not available.

**Table 2:** Dysregulated lncRNAs and their roles in HCC.

Name	Expression	Targets	Action Modes	Pathways	Outcomes	Ref.
*SNHG5*	Up	UPF1	DNA binding	Wnt/β-catenin pathway	Proliferation (+)	[149]
*uc.134*	Down	CUL4A	DNA binding	Ubiquitination and phosphorylation pathway	Proliferation (+)	[150]
*ASH1L-AS1*	Up	ERK1/2	Encodes proteins	MAPK signaling pathway	Proliferation (+), metastasis (+), invasion (+)	[140]
*PWRN1*	Down	PKM2	Enzyme binding	Glycolysis pathway	Proliferation (−)	[151]
*AIRN*	Up	STAT1	Regulation of protein stability	Ubiquitination of STAT1 protein pathway	Proliferation (+), apoptosis (−)	[152]
*HULC*	Up	PTEN	Upregulation of sirt1 and beclin-1 and downregulate miR15a	AKT/PI3K/mTOR pathway	Proliferation (+)	[153]
*SZT2-AS1*	Up	SMYD2	Recruitment of methyltransferase SMYD2	Hypoxia pathway	Proliferation (+), metastasis (+), angiogenesis (+)	[154]
*NEAT1*	Up	miR-362-3p	Competitive binding to miR-362-3p	Ferroptosis pathway	Proliferation (−), apoptosis (+), metastasis (−)	[155]
*H19*	Up	miR-200b-3p, PPP1CA	miRNA sponging and binding with enzyme	MAPK pathway	Migration (+), invasion (+)	[156]
*SNHG5*	Up	GSK3β	Competitive binding to miR-26a-5p	Wnt/β-catenin pathway	Proliferation (+), metastasis (+)	[157]
*FTO-IT1*	Up	FTO	Interaction with protein complex and regulation of mRNA stability	Glycolysis pathway	Proliferation (+), metastasis (+), invasion (+)	[158]

*ASH1L*, absent, small, or homeotic 1-like; *AIRN*, antisense of Igf2r nonprotein coding RNA; *HULC*, highly upregulated liver cancer; *FTO-IT1*, target fat mass and obesity-associated intronic transcript 1; *NEAT1*, nuclear-enriched abundant transcript 1; *PWRN1*, Prader–Willi region nonprotein coding RNA 1; *SNHG5*, small nucleolar RNA host gene 5; *SZT2-AS1*, seizure threshold 2 antisense RNA 1.

## Data Availability

No new data was generated or analyzed in this study. The information discussed in this review is based on previously published studies, which are cited in the manuscript.

## Abbreviations

The following abbreviations are used in this manuscript:

HCC	Hepatocellular Carcinoma
HBV	Hepatitis B Virus
HCV	Hepatitis C Virus
HBx	Hepatitis B Virus xProtein
MASLD	Metabolic Dysfunction-Associated Steatotic Liver Disease
CHB	Chronic Hepatitis B
ALD	Alcohol-Associated Liver Disease
AFP	Alpha-Fetoprotein
BCLC	Barcelona Clinic Liver Cancer
EMT	Epithelial–Mesenchymal Transition
PTM	Post-Translational Modification
ncRNA	Noncoding RNA
miRNA	MicroRNA
lncRNA	Long Noncoding RNA
m^6^A	N6-Methyladenosine
mRNA	Messenger RNA
cfDNA	Cell-Free DNA
ctDNA	Circulating Tumor DNA
DNMT	DNA Methyltransferase
CpG	Cytosine-Phosphate-Guanine
HDAC	Histone Deacetylase
HAT	Histone Acetyltransferase
HMTases	Histone Methyltransferase
EZH2	Enhancer of Zeste Homolog 2
BET	Bromodomain and Extra-Terminal
BRD4	Bromodomain Containing Protein 4
EVs	Extracellular Vesicles
OS	Overall Survival
ICIs	Immune Checkpoint Inhibitors
